# Advanced electrocardiography heart age: a prognostic, explainable machine learning approach applicable to sinus and non-sinus rhythms

**DOI:** 10.1093/ehjdh/ztae075

**Published:** 2024-10-09

**Authors:** Zaidon S Al-Falahi, Todd T Schlegel, Israel Palencia-Lamela, Annie Li, Erik B Schelbert, Louise Niklasson, Maren Maanja, Thomas Lindow, Martin Ugander

**Affiliations:** Kolling Institute, Royal North Shore Hospital, University of Sydney, St Leonards, Sydney, NSW 2065, Australia; Department of Cardiology, Campbelltown Hospital, South West Sydney Local Health District, NSW 2560, Australia; Department of Clinical Physiology, Karolinska University Hospital, and Karolinska Institutet, SE-17176 Stockholm, Sweden; Nicollier-Schlegel SARL, Trélex 1270, Switzerland; Kolling Institute, Royal North Shore Hospital, University of Sydney, St Leonards, Sydney, NSW 2065, Australia; Kolling Institute, Royal North Shore Hospital, University of Sydney, St Leonards, Sydney, NSW 2065, Australia; Minneapolis Heart Institute East, United Hospital, Minneapolis, MN 55407, USA; Minneapolis Heart Institute East, United Hospital, Minneapolis, MN 55407, USA; Department of Clinical Physiology, Karolinska University Hospital, and Karolinska Institutet, SE-17176 Stockholm, Sweden; Kolling Institute, Royal North Shore Hospital, University of Sydney, St Leonards, Sydney, NSW 2065, Australia; Department of Medicine, Research and Development, Växjö Central Hospital, 35188 Region Kronoberg, Sweden; Respiratory Medicine, Allergology and Palliative Medicine, Clinical Sciences, Lund University, 22100 Lund, Sweden; Kolling Institute, Royal North Shore Hospital, University of Sydney, St Leonards, Sydney, NSW 2065, Australia; Department of Clinical Physiology, Karolinska University Hospital, and Karolinska Institutet, SE-17176 Stockholm, Sweden

**Keywords:** ECG, Accelerated aging, Advanced ECG analysis, Machine learning, Risk prediction

## Abstract

**Aims:**

An explainable advanced electrocardiography (A-ECG) Heart Age gap is the difference between A-ECG Heart Age and chronological age. This gap is an estimate of accelerated cardiovascular aging expressed in years of healthy human aging, and can intuitively communicate cardiovascular risk to the general population. However, existing A-ECG Heart Age requires sinus rhythm. We aim to develop and prognostically validate a revised, explainable A-ECG Heart Age applicable to both sinus and non-sinus rhythms.

**Methods and results:**

An A-ECG Heart Age excluding P-wave measures was derived from the 10-s 12-lead ECG in a derivation cohort using multivariable regression machine learning with Bayesian 5-min 12-lead A-ECG Heart Age as reference. The Heart Age was externally validated in a separate cohort of patients referred for cardiovascular magnetic resonance imaging by describing its association with heart failure hospitalization or death using Cox regression, and its association with comorbidities. In the derivation cohort (*n* = 2771), A-ECG Heart Age agreed with the 5-min Heart Age (*R*^2^ = 0.91, bias 0.0 ± 6.7 years), and increased with increasing comorbidity. In the validation cohort [*n* = 731, mean age 54 ± 15 years, 43% female, *n* = 139 events over 5.7 (4.8–6.7) years follow-up], increased A-ECG Heart Age gap (≥10 years) associated with events [hazard ratio, HR (95% confidence interval, CI) 2.04 (1.38–3.00), C-statistic 0.58 (0.54–0.62)], and the presence of hypertension, diabetes mellitus, hypercholesterolaemia, and heart failure (*P* ≤ 0.009 for all).

**Conclusion:**

An explainable A-ECG Heart Age gap applicable to both sinus and non-sinus rhythm associates with cardiovascular risk, cardiovascular morbidity, and survival.

## Graphical Abstract

**Graphical Abstract:**
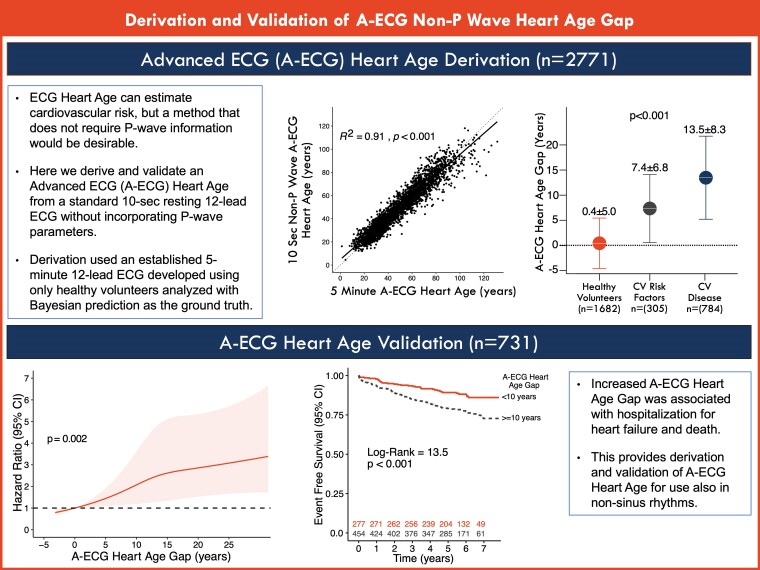

## Introduction

Primary prevention in cardiovascular disease has underpinned the reduction in cardiovascular death over the decades and stands out as one of the most effective strategies for reducing its burden.^1–3^ Guidelines generally encourage both systematic and opportunistic cardiovascular screening with a focus on high-risk individuals.^2,4,5^ Despite being the simplest and most accessible cardiovascular diagnostic modality, current guidelines advise against the use of the traditional 12-lead electrocardiogram (ECG) in population screening due to its limited diagnostic performance.^6^ However, the ECG is known to contain clinically relevant information that is neither visually apparent nor immediately extractable with strictly conventional ECG analysis methods.^7^ Advanced ECG (A-ECG) analysis is an explainable method of extracting such information from the ECG that offers markedly improved diagnostic performance over conventional ECG analysis, and could potentially enable more effective pre-symptomatic screening.^8^ In contrast to other published deep learning-based methods for age estimation,^9,10^ A-ECG is built using transparent supervised machine learning techniques including Bayesian statistics, multivariable regression^11^ and singular value decomposition.^12^ An important contrasting feature is that A-ECG Heart Age was not developed to predict an individual’s chronological age, but rather to estimate the deviation from chronological aging, especially accelerated aging caused by cardiovascular risk factors and disease.

A-ECG analysis using high-fidelity 5-min ECG recordings has been used to develop an accurate Bayesian estimation of Heart Age concordant with the chronological age in strictly healthy volunteers. ECG Heart Age by this method incrementally deviates from chronological age in individuals with cardiovascular risk factors and those with established cardiovascular disease.^13^ The reference 5-min Bayesian A-ECG Heart Age was used to derive a 10-s A-ECG Heart Age applicable to standard 12-lead ECGs used in clinical practice. A-ECG Heart Age gap was then calculated as the difference between A-ECG Heart Age and chronological age; an increasing Heart Age gap has been shown to be associated with cardiovascular risk factors, heart failure and mortality.^14,15^ However, the previously presented A-ECG Heart Age estimation relied on the presence of quantifiable P-waves, limiting its utility to individuals in sinus rhythm. We hypothesized that Heart Age could also be derived using a standard 10-s ECG without including P-wave information. We therefore aimed to evaluate whether 10-s A-ECG could reliably predict Heart Age as compared with the 5-min A-ECG while excluding all P-wave related parameters. We also aimed to externally validate the prognostic performance of the Heart Age gap estimation by determining the association with conventional risk factors and heart failure hospitalization or death.

It is worth noting that this study aims primarily to derive an A-ECG Heart Age that can be applied to non-sinus rhythm and validates it internally and externally against previously published cohorts. Testing the non-P A-ECG Heart Age in cohorts with non-sinus rhythms is a separate research focus and is currently the subject of active research.

In summary, this paper contributes to the existing literature in the following manner:

1. The study derives and provides prognostic validation for a new A-ECG Heart Age that can be used for cardiovascular risk stratification, and that can be universally applied to both sinus and non-sinus rhythms.
2. The study provides mechanistic and prognostic demonstration that the ECG encodes age related information, including evidence of accelerated aging, in domains beyond the P-wave.

## Methods

Two separate cohorts were used for this study, a derivation cohort and a separate validation cohort. For derivation, ECGs from 2771 individuals (1682 healthy volunteers, 305 individuals with cardiovascular risk factors, and 784 patients with established cardiovascular disease) were used.^8,13^ The validation cohort consisted of ECGs from patients who had undergone clinical cardiac magnetic resonance imaging (CMR) imaging at University of Pittsburgh Medical Centre (UPMC, Pittsburgh, PA, USA).

### Heart age estimation in the derivation cohort

This derivation cohort was recruited through collaboration between NASA, the USA-Slovenia Cooperation in Science and Technology, the McDonald Fund of St. Luke’s Episcopal Hospital in Houston, the Charleston Cardiology Research Foundation, the University of the Andes Research Fund, the Swedish National Health Service, Swedish Research Council, Swedish Heart Lung Foundation and Lund University Medical Faculty. These included data from:

1. Cardiac clinic patients who volunteered for individual studies at any of the following clinical sites: Texas Heart Institute (Houston, TX, USA); the University of Texas Medical Branch (Galveston, TX, USA); the University of Texas Health Sciences Center (San Antonio, TX, USA); Brooke Army Medical Center (San Antonio, TX, USA); St. Francis Hospital (Charleston, WV, USA); the Universidad de los Andes (Mérida, Venezuela); and Lund University Hospital (Lund, Sweden); and
2. Asymptomatic individuals who volunteered as controls subjects at any of the following sites: Johnson Space Center (Houston, TX, USA); the Universidad de Los Andes (Merida, Venezuela) and Lund University Hospital (Lund, Sweden).

Individuals included in the healthy cohort were low risk, asymptomatic volunteers with no identifiable cardiovascular or systemic disease based on clinical history and physical examination. They had a body mass index of 26 ± 4 kg/m^2^. Furthermore, active smokers, those with increased blood pressure at physical examination (≥ 140/90 mm Hg), and those on treatment for hypertension or diabetes were excluded from the healthy cohort.

Within the derivation cohort, assignment to the established cardiovascular disease group was based on the presence of any of the following:

1. Coronary heart disease, determined by coronary angiography with at least one obstructed vessel (≥ 50%) in at least one major coronary vessel, history of coronary artery bypass, or alternatively one or more reversible perfusion defects on ^99m^Tc-tetrofosmin single-photon emission computed tomography (SPECT);
2. Left ventricular hypertrophy (LVH) based on imaging evidence of at least moderate, concentric wall thickening according to guidelines of the American Society of Echocardiography;
3. Left ventricular systolic dysfunction (left ventricular ejection fraction (LVEF) ≤ 50%) at echocardiography, CMR imaging or SPECT, with findings suggestive of ischaemic or non-ischaemic cardiomyopathy; or
4. Echocardiographic or CMR-related findings consistent with hypertrophic cardiomyopathy.

Notably, the A-ECG features used in Heart Age were derived using strictly the healthy volunteers. The group with established cardiovascular disease were used only to optimize the model fit. Further details of the derivation cohort have been previously published.^8,13^ Institutional Review Board (IRB) approvals were obtained from NASA’s Johnson Space Centre and partner hospitals that fall under IRB exemptions for previously collected and de-identified data.^8^

All individuals in the derivation cohort underwent both 5-min and 10-s A-ECG analysis within 1 month of clinical diagnostic imaging, and the investigators performing the A-ECG analysis were blinded to the results of diagnostic clinical imaging. Within the strictly healthy cohort, A-ECG parameters were derived from 5-min ECGs using specialized software developed by TT Schlegel *et al.*^16,17^ to derive parameters for:

1. 12-lead High Frequency QRS ECG^16^
2. Derived 3D ECG using the Frank-lead reconstruction technique suggested by Kors *et al.*^18^
3. QRS and T-waveform complexity via singular value decomposition to derive such measures as the principal component analysis ratio, the relative residuum, and the dipolar and non-dipolar voltage equivalents of the QRS and T waveforms.^17,19–22^
4. Variability analyses derived measures for R–R and QT variability.^23–26^

A comprehensive list of all the A-ECG parameters considered for the A-ECG Heart Age model is provided as Supplementary material online, *Supplement 1*. Using the variables derived from 5-min A-ECG, known chronological age and the confirmed state of health, Heart Age was estimated using a Bayesian approach. For a detailed discussion of the mathematical approaches used, refer to the work of Ball *et al.*^13^ The estimated 5-min A-ECG Heart Age is thus set as the ground truth for the purpose of deriving a standard 10-s ECG Heart Age without P-wave related measures that could be applied in clinical practice to individuals in non-sinus rhythm.

To derive a 10-s A-ECG heart age excluding P-wave information, multiple steps were required. Initially, A-ECG parameters from 10-s ECG were obtained for individuals in the same healthy derivation cohort. Standard least squares linear regression was then used to identify parameters most predictive of the reference 5-min A-ECG Heart Age, and over 20 such parameters with a *P*-value of < 0.0001were identified.

**Figure 1:**
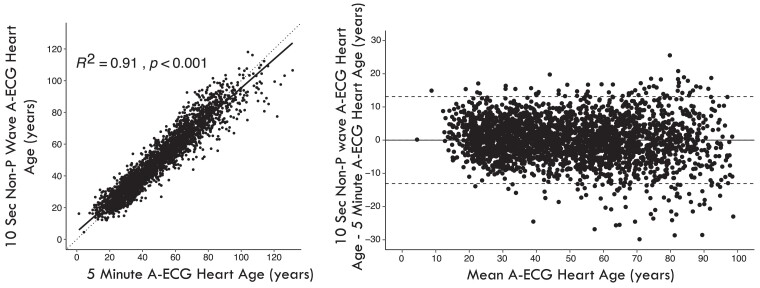
Left panel: scatter plot showing the relationship between the 10-s, A-ECG Heart Age and the 5-min A-ECG Heart Age in the derivation cohort. The *R*^2^ value was 0.91 (*P* < 0.001). Right panel: Bland–Altman plot showing the difference between the 10-s A-ECG Heart Age and 5-min A-ECG Heart Age in relation to the mean of both ECG heart ages. The agreement between methods is strong, with minimal deviation from the identity line (dashed) or bias (0.0 ± 6.7 years).

Subsequently, stepwise multiple linear regression was conducted on these parameters to create a multivariable model that best predicted the 5-min ECG Heart Age in the healthy group, while excluding all P-wave related measures. The chosen optimal model was the simplest one that achieved the highest *R*^2^ value and had *P*-values of < 0.0001 for the individual measures in the healthy group and < 0.01 when applied to the wider cohort with cardiovascular risk factors or disease. The final optimal non-P Wave 10-s A-ECG Heart Age model showed an excellent correlation with the reference 5-min A-ECG model (*R*^2^ = 0.91, *P* < 0.0001) (*Figure 1*).

### External validation of the non-P-wave Heart Age Gap

A separate cohort was used for the purpose of validating the prognostic value of the A-ECG Heart Age gap described above. This validation cohort consisted of ECGs from patients who had undergone clinical CMR imaging at University of Pittsburgh Medical Centre (UPMC). All patients had 12-lead 10-s ECG recorded within 30 days of the CMR exam and follow-up for the available endpoints of death or hospitalization for heart failure. In the validation cohort, patients with missing follow-up data, heart rate ≥ 100/min, QRS duration ≥ 130 ms, atrial fibrillation or flutter, or digoxin use were excluded from the analysis. The validation cohort has been described in previous studies.^27,28^ For the validation cohort, approval was obtained from the IRB at University of Pittsburgh Medical Centre. Written consent was obtained for all participants for both cohorts, and all data was analysed following de-identification.

### Statistical analysis

Continuous variables were described using either mean ± SD or median (interquartile range). The *χ*^2^ test was used to test for proportional differences between groups. When deriving the 10-s A-ECG Heart Age score, variable selection was determined in the healthy cohort through stepwise standard least squares multivariable linear regression for the prediction of the Bayesian 5-min Heart Age as the reference standard. Selection of variables was based on achieving the most parsimonious multivariable- model with the highest possible model *R*^2^ with individually statistically significant (*P* < 0.0001) measures. The intercept and coefficients of the prediction model was subsequently determined after applying the final model to both healthy subjects, patients with cardiovascular risk factors, and established cardiovascular disease using the 5-min Bayesian A-ECG Heart Age as the reference standard.

Time-to-event analysis was conducted in the validation cohort, and Kaplan–Meier curves were constructed with censoring at the study’s end. The subjects were divided into two categories based on their Heart Age gap: those with a gap of < 10 years and those with a gap of 10 years or more, where 10 years approximately represents two Sds from the average Heart Age gap among healthy subjects in the initial derivation cohort.^14^ The association between the A-ECG Heart Age gap and a composite endpoint of hospitalization for heart failure or death was analysed using Cox proportional hazard regression, unadjusted and adjusted for potentially confounding covariates of age, sex, and cardiovascular risk factors (smoking, diabetes mellitus, hypertension, hypercholesterolaemia, and body mass index). A potential interaction effect between age and Heart Age gap on the association with outcomes was evaluated by analysing models with and without an interaction term for age (heart age gap × age). These models were then compared using the likelihood ratio test. HRs are presented with 95% CIs for each 5-year increment in the Heart Age gap. A restricted cubic spline regression model was used to demonstrate risk increments with increasing Heart Age gap. Repeatability was assessed by describing the minimum detectable change (1.96 × √2 × standard error of the mean difference) in Heart Age gap 1 year later in a subset of healthy patients (*n* = 27) for both the P-wave inclusive and P-wave exclusive Heart Age estimation. These patients all remained healthy at the time of the repeat ECG recording.^29^ These results are presented in Supplements, Supplementary material online, *Table S1*. Regression analysis and the derivation of the A-ECG Heart Age model were performed using SAS JMP version 11.0 (SAS Institute Inc., Cary, NC, USA). All other analyses were performed using the software R (version 4.2.2, R Core Team, R Foundation for Statistical Computing, Vienna, Austria).

## Results

### Derivation of the A-ECG Heart Age

**Figure 2:**
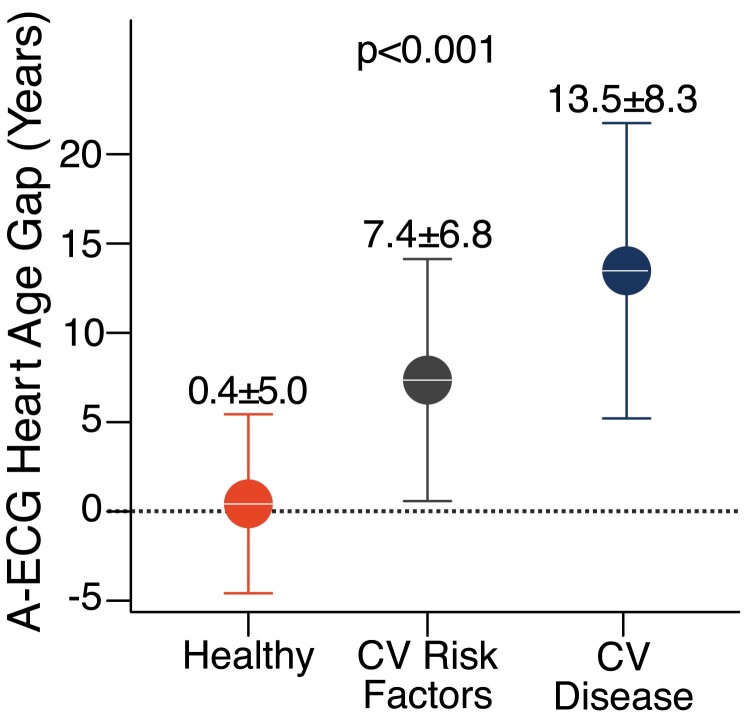
A-ECG Heart Age gap in healthy individuals (left, orange), individuals with cardiovascular (CV) risk factors (middle, dark grey), and patients with CV disease (right, navy blue). On average, there is a negligible difference between Heart Age and chronological age in healthy individuals, whereas the gap is increased in individuals at CV risk and highest for those with overt CV disease.

Characteristics of the derivation cohort are summarized in *Table 1*. The final A-ECG Heart Age model with its 10 included measures is presented with intercepts and coefficients in *Tables 2* and *3* for males and females, respectively. No P-wave related information is included in the model. The slightly different measures between males and females likely stem from inherent differences in cardiac repolarization, body habitus and hormonal effects between the two sexes.^30^ The mean values for the included A-ECG measures are summarized in *Table 4*, stratified by state of health, risk factors, and disease. The agreement between the Heart Age and the 5-min Heart Age reference standard was strong (*R*^2^ = 0.91, bias 0.0 ± 6.7 years, *Figure 1*). In healthy individuals, the Heart Age gap was 0.4 ± 5.0 years. In individuals with risk factors it was 7.4 ± 6.8 years, and among those with established cardiovascular disease it was 13.5 ± 8.3 years (*Figure 2*).

**Table 1:** Derivation cohort characteristics

	All (*n* = 2771)	Healthy (*n* = 1682)	CV risk factors (*n* = 305)	CV disease (*n* = 784)
Age (years)	46.2 ± 16.0	38.6 ± 13.0	54.8 ± 11.2	59.0 ± 13.2
Male sex, *n* (%)	1645 (59.4)	999 (59.4)	163 (53.4)	483 (61.6)
Heart age gap (years)	4.9 ± 8.6	0.4 ± 5.0	7.4 ± 6.8	13.5 ± 8.3
CAD, *n* (%)	421 (15.2)	–	–	421 (53.7)
ICM, *n* (%)	120 (94.3)	–	–	120 (15.3)
HCM, *n* (%)	92 (3.3)	–	–	92 (11.7)
LVH, *n* (%)	96 (3.5)	–	–	96 (12.2)
NICM, *n* (%)	53 (1.9)	–	–	53 (6.8)

CAD, coronary artery disease; HCM, hypertrophic cardiomyopathy; ICM, ischaemic cardiomyopathy; LVH, left ventricular hypertrophy; NICM, non-ischaemic cardiomyopathy.

**Table 2:** A-ECG measures included in the Heart Age for males

Measure	Coefficient	*t* ratio
Intercept	−166.22	12
Chronological age (years)	0.900	99
Ln of corrected spatial QT interval (ms)	27.5	13
Ln of the ‘Intradipolar ratio’ of T-wave complexity from the Frank XYZ leads [(T Eigenvalue2 × T Eigenvalue3)/(T Eigenvalue1)^2^] after singular value decomposition, Ln (%)	1.69	12
R-wave amplitude in derived lead Y (µV)	−0.00740	−12
Corrected spatial JT interval (ms)	0.0580	11
Spatial T-wave axis (sine radians)	−3.41	−9
Max amplitude of the derived VCG frontal planar QRS loop (µV)	0.00302	6
Mean difference between the frontal plane T-wave elevation and azimuth of all T-wave loop samples (degrees)	0.0279	5

Ln, natural logarithm; VCG, vectorcardiographic.

**Table 3:** A-ECG measures included in the Heart Age for females

Measure	Coefficient	*t* Ratio
Intercept	−200	
Chronological age (years)	0.942	91
VCG frontal plane QRS axis (sine radians)	−9.59	−23
Gender	4.20	14
Natural logarithm Ln of corrected spatial QT interval (ms)	32.3	13
R-wave amplitude in lead Y (µV)	−0.00869	−12
Ln of the ‘Intradipolar ratio’ of T-wave complexity from the Frank XYZ leads [(T Eigenvalue2 × T Eigenvalue3)/(T Eigenvalue1)^2^] after singular value decomposition, Ln (%)	1.88	12
Corrected spatial JT interval (ms)	0.0676	11
Spatial T-wave axis (sine radians)	−3.77	−9
Max amplitude of the derived VCG frontal planar QRS loop (µV)	0.00345	6
Mean difference between the frontal plane T-wave elevation and azimuth of all T-wave loop samples (degrees)	0.0334	5

Ln, natural logarithm; VCG, vectorcardiographic.

**Table 4:** A-ECG parameters in the derivation cohort stratified by health and presence of cardiovascular risk factors or established cardiovascular disease

	Healthy	Risk factors	Disease	*P*
*n* (%)	1682 (60.7)	305 (11)	784 (28.3)	–
Chronological age (years)	39 ± 13	55 ± 11	59 ± 13	< 0.001
R-wave amplitude in lead Y (μV)	1144 ± 392	828 ± 363	717 ± 462	< 0.001
VCG frontal plane QRS axis (sine radians)	0.82 ± 0.23	0.54 ± 0.44	0.29 ± 0.54	< 0.001
Max amplitude of the derived VCG frontal planar QRS loop (μV)	1617 ± 411	1384 ± 385	1286 ± 599	< 0.001
Ln of the ‘Intradipolar ratio’ of T-wave complexity from the Frank XYZ leads [(T Eigenvalue2 × T Eigenvalue3)/(T Eigenvalue1)^2^] after singular value decomposition, Ln (%)	−0.69 ± 0.83	−0.28 ± 0.89	0.43 ± 1.12	< 0.001
Ln of the corrected spatial QT interval (ms)	6.00 (0.06)	6.05 (0.06)	6.07 (0.08)	< 0.001
Spatial T-wave axis (sine radians)	0.71 (0.20)	0.63 (0.28)	0.46 (0.54)	< 0.001
Corrected spatial JT interval (ms)	336 ± 23	355 ± 29	364 ± 37	< 0.001
Mean difference between the frontal plane T-wave elevation and azimuth of all T-wave loop samples (degrees)	41 ± 16	47 ± 22	72 ± 36	< 0.001

Ln, natural logarithm; VCG, vectorcardiographic.

### External validation of the Heart Age gap

The validation cohort included 731 patients with a median follow-up of 5.7 (4.8–6.7) years, during which 138 (18.9%) individuals experienced the primary combined outcome of hospitalization for heart failure or death [101 (13.8%) deaths, 57 (7.8%) hospitalizations for heart failure, and 20 (2.7%) both hospitalization and death]. Baseline characteristics of the validation cohort are presented in *Table 5*.

**Table 5:** Validation cohort detailed characteristics

	All cohort	Heart age gap (years)		
		< 10	≥ 10	*P*
Number of individuals, *n* (%)	731 (100)	277 (37.9)	454 (62.1)	
Chronological age (years)	53.6 ± 15.3	49.6 ± 15.2	56.0 ± 14.8	< 0.001
A-ECG Heart Age (years)	66.7 ± 19	54.4 ± 16.1	74.1 ± 16.7	< 0.001
A-ECG Heart Age gap (years)	13.1 ± 8.2	4.8 ± 3.8	18.1 ± 5.7	< 0.001
Male sex, *n* (%)	417 (57.0)	160 (57.8)	257 (56.6)	0.82
Body mass index (kg/m^2^)	30 ± 7.8	29.4 ± 7.6	30.3 ± 7.8	0.12
Body surface area (m^2^)	2.0 ± 0.3	2.0 ± 0.3	2.1 ± 0.3	0.29
All-cause mortality, *n* (%)	101 (13.8)	28 (10.1)	73 (16.1)	0.031
Death or HF hospitalization, *n* (%)	138 (18.9)	34 (12.3)	104 (22.9)	0.001
NT-proBNP (ng/L)	64 [28–188]	43 [20–131]	82 [33–222]	< 0.001
eGFR, mL/min/1.73 m^2^	89 ± 25	93 ± 23	86 ± 26	0.001
Hypertension, *n* (%)	379 (51.8)	120 (43.3)	259 (57.0)	< 0.001
Diabetes mellitus, *n* (%)	157 (21.5)	33 (11.9)	124 (27.3)	< 0.001
Hyperlipidaemia, *n* (%)	295 (40.4)	86 (31.0)	209 (46.0)	< 0.001
Chronic kidney disease, *n* (%)	18 (2.5)	2 (0.7)	16 (3.5)	0.034
Smoker, *n* (%)	119 (16.3)	50 (18.1)	69 (15.2)	0.36
CABG, *n* (%)	56 (7.7)	20 (7.2)	36 (7.9)	0.84
PCI, *n* (%)	89 (12.2)	25 (9.0)	64 (14.1)	0.06
Heart failure hospitalizations, *n* (%)	57 (7.8)	11 (4.0)	46 (10.1)	0.004
HFrEF, *n* (%)	166 (22.7)	48 (17.3)	118 (26.0)	0.009
HFpEF, *n* (%)	51 (7)	13 (4.7)	38 (8.4)	0.081
Cardiovascular magnetic resonance imaging parameters				
End-diastolic LV volume (mL)	172 ± 63	167 ± 57	175 ± 67	0.084
End-systolic LV volume (mL)	83 ± 58	75 ± 47	85 ± 63	0.003
LVEF (%)	55 ± 14	57 ± 12	54 ± 15	0.002
GLS (%)	−16 ± 4	−16 ± 4	−15 ± 5	< 0.001
LV mass (g)	120 ± 46	115 ± 42	124 ± 49	0.009
Presence of LGE (%)	263 (36)	83 (30.0)	180 (39.6)	0.010
Extracellular volume (%)	27.9 (3.8)	27.4 (3.5)	28.2 (4)	0.008
Medications				
Insulin, *n* (%)	107 (14.6)	23 (8.3)	84 (18.5)	< 0.001
Oral hypoglycaemic, *n* (%)	49 (6.7)	12 (4.3)	37 (8.1)	0.06
ACE/ARB, *n* (%)	295 (40.4)	110 (39.7)	185 (40.7)	0.84
No medications, *n* (%)	133 (18.2)	73 (26.4)	60 (13.2)	< 0.001
Beta blockers, *n* (%)	359 (49.1)	109 (39.4)	250 (55.1)	<0.001
Loop diuretics, *n* (%)	142 (19.4)	39 (14.1)	103 (22.7)	0.006
Calcium channel blocker, *n* (%)	55 (7.5)	14 (5.1)	41 (9.0)	0.067
Hydrochlorothiazide, *n* (%)	68 (9.3)	22 (7.9)	46 (10.1)	0.39
Nitroglycerin, *n* (%)	23 (3.1)	6 (2.2)	17 (3.7)	0.33
Statin, *n* (%)	301 (41.2)	91 (32.9)	210 (46.3)	< 0.001
Warfarin, *n* (%)	45 (6.2)	14 (5.1)	31 (6.8)	0.42
Antiplatelets, *n* (%)	379 (51.8)	120 (43.3)	259 (57.0)	< 0.001

ACE, angiotensin-converting enzyme; ARB, angiotensin receptor blockers; CABG, coronary artery bypass grafting; CAD, coronary artery disease; eGFR, estimated glomerular filtration rate; GLS, global longitudinal strain; HF, heart failure; HFrEF, heart failure with reduced ejection fraction; HFpEF, heart failure with preserved ejection fraction; LGE, late gadolinium enhancement; LV, left ventricle; LVEF, left ventricular ejection fraction; NT-proBNP, N-terminal prohormone of brain natriuretic peptide; PCI, percutaneous coronary intervention.

Cardiovascular risk factors and diseases were more prevalent among patients with a higher gap (≥ 10 years) compared with those with a lower gap (< 10 years; hypertension 57 vs. 43%, *P* < 0.001; diabetes 27 vs. 12% *P* < 0.001; hyperlipidaemia 46 vs. 31%, *P* < 0.001). In addition, abnormal CMR imaging findings were more prevalent among those with higher gap, compared with those with a lower gap (lower LVEF, worse global longitudinal strain, higher left ventricular mass, greater presence of late gadolinium enhancement, and higher myocardial extracellular volume, *P* < 0.01 for all).

**Figure 3:**
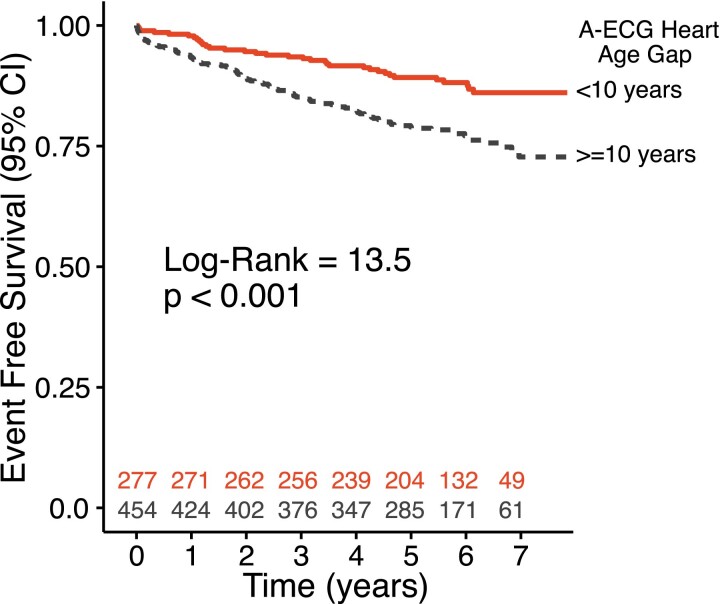
Time-to-event analysis for individuals with A-ECG Heart Age gap < 10 years (dense line) vs. those with an A-ECG Heart Age gap≥10 years (dashed line) for hospitalization for heart failure or death in the external validation cohort.

**Figure 4:**
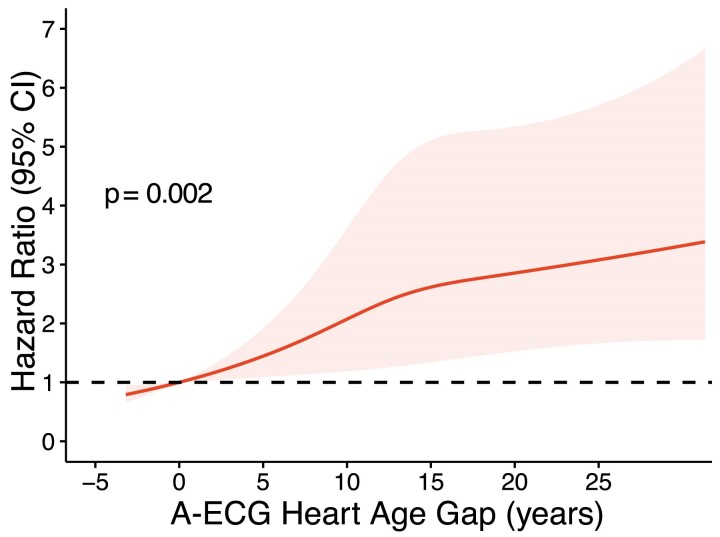
Restricted cubic splines plot showing the continuously increasing HR for death or hospitalization for heart failure with increasing A-ECG heart age gap. The solid line indicates the HR with the shaded area indicating the 95% confidence interval, the dashed line is set at a HR of 1.

Increased gap (≥ 10 years) was associated with increased risk of heart failure admission or death [unadjusted HR: 2.04 (1.38–3.00), adjusted for age and sex: 1.55 (1.04–2.30)] (*Figures 3* and *4*). The interaction test for gap and age did not achieve statistical significance (*P* = 0.06). Given the close proximity to the pre-defined significance level (*P* < 0.05), HRs were also calculated after stratifying data by age. Heart age gap ≥ 10 years was associated with heart failure or death among younger individuals [< 60 years: HR 2.98 (1.68–5.30)], but not among individuals ≥ 60 years of age [HR 1.01 (0.60–1.71)].

**Figure 5:**
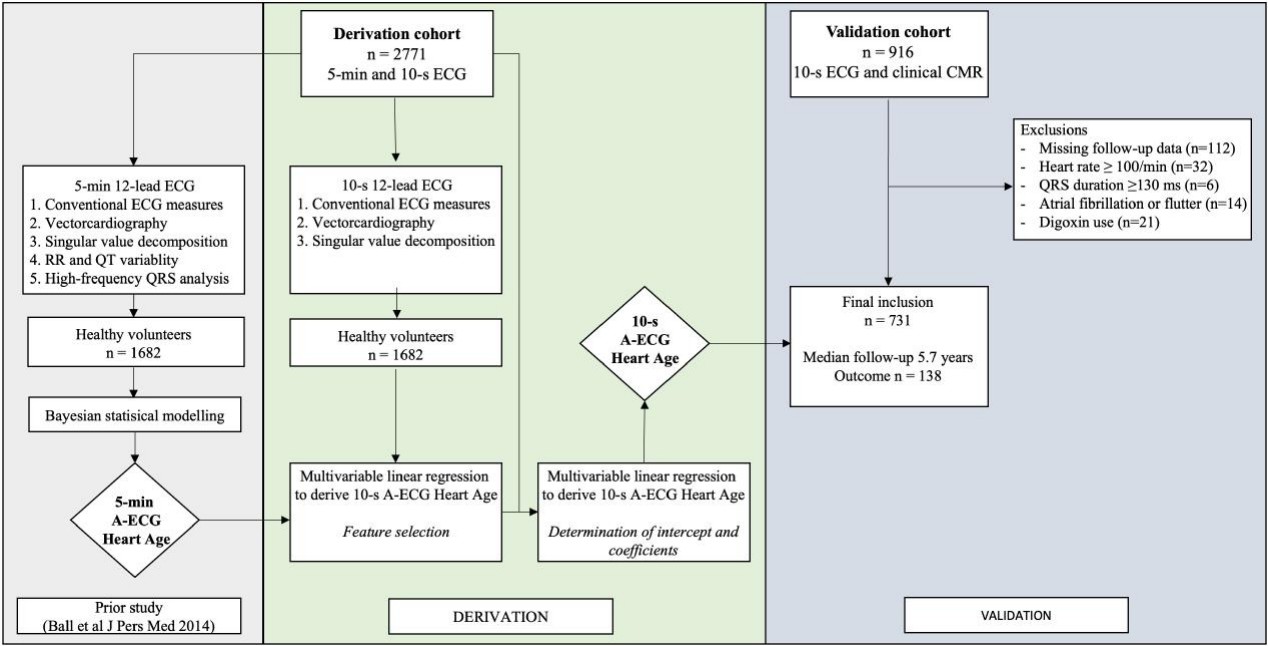
Workflow summary of 5-min A-ECG Heart Age and 10-s A-ECG Heart Age derivation and validation.

*Figure 5* is a workflow diagram summarizing the steps of 10-s A-ECG Heart Age derivation from the 5-min A-ECG Heart Age and its validation.

## Discussion

The main finding of this study is that an estimation of A-ECG Heart Age containing useful prognostic value can be performed without the use of P-wave information. Healthy volunteers had a Heart Age similar to their chronological age, i.e. their gap was close to zero. In comparison, individuals with cardiovascular risk factors had a gap of ∼7 years, while those with established disease had a gap of 14 years. In the external validation cohort, high gap was associated with higher prevalence of cardiovascular risk factors, abnormal CMR findings, and ultimately hospitalization for heart failure or death.

The current study builds on prior work enabling the use of an A-ECG Heart Age on standard 10-s, 12-lead ECGs rather than a 5-min, 12-lead higher-fidelity ECG as originally devised.^13–15^ However, P wave duration constituted an important part of the initial 10-s A-ECG model for Heart Age, limiting its use to individuals in sinus rhythm.^14^ The current study therefore now extends the applicability of 10-s A-ECG Heart Age estimations to patients with atrial fibrillation or other causes of non-quantifiable P-waves.

The current study mechanistically shows that determinants of the emerging concept of cardiovascular aging are embedded in the ECG along domains beyond the P-wave, which has previously been described as an important measure associated with cardiovascular aging and associated pathologies.^31–34^ In the original 10-s A-ECG Heart Age gap, P-wave and spatial QT durations were the two most important A-ECG measures in the model. In contrast, in the Heart Age gap of the current study, the axis of the spatial QRS axis in the derived vectorcardiographic frontal plane and the heart rate-corrected spatial QT interval were the two most important ECG measures in the model. Indeed, the normal evolution of these two metrics with aging is well known.^35,36^

Higher Heart Age gap was also associated with both adverse outcomes and underlying comorbidities. This is consistent with multiple recent studies on the concept of biological age estimation using the ECG.^9,10,31,37–41^ For example, a deep neural network (DNN) has been trained to predict sex and age from the 12-lead ECG. In that study, those with a predicted Heart Age > 7 years higher than their chronological age (gap ≥ 7 years) had a higher prevalence of coronary disease, hypertension, and reduced LVEF, while those with a lower gap had fewer events in long-term follow-up.^9^ Similarly, DNN-estimated ECG age trained on a large cohort (*n* = 1 558 415) showed increased risk with increasing gap.^10^

A-ECG emphasizes the use of transparently explainable and quantifiable ECG measures for Heart Age estimation. This differs from other attempts at Heart Age estimation using the ECG, particularly from DNN-type modelling. For example, DNN techniques continue to have the inherent limitation of model opaqueness and non-explainability and as such are still considered ‘black box’ methods. In the only head-to-head comparison on an identical external validation cohort, the original 10-second A-ECG Heart Age outperformed a DNN-based Heart Age estimation model in prognostic strength.^15^ The A-ECG Heart Age estimation derived from the current work showed similar associations with survival as the original A-ECG Heart Age, as demonstrated in the identical validation population. Thus, when to the DNN-based estimation, the currently proposed A-ECG Heart Age method shows a superior performance for outcomes.

In terms of explainability, measures included in the Heart Age gap are transparently quantifiable digital biomarkers of cardiac electrophysiology. Some of these digital biomarkers have been described extensively in the literature, such as the frontal plane QRS axis, spatial QT duration, vectorcardiographic T-wave axis, spatial ventricular gradient and certain ratios between the T-wave eigenvalues that quantify the complexity of the T-wave in 3D space. Furthermore, the deviations of these measures from normal ranges are known to be associated with adverse events.^19,42–47^ In contrast, DNN models are more difficult to explain, and it has been shown that their performance can be impacted by such technical factors as sampling rate, signal duration, and data augmentation.^48^ A-ECG Heart Age uses established ECG features that are quantified by measures that are, anecdotally, less prone to these factors.

In this A-ECG Heart Age model, chronological age was included and used as the foundation from which accelerated or decelerated cardiac aging can be quantified based on electrocardiographic deviations from healthy aging. For example, if a given ECG measure increases with healthy aging, it is only by incorporating chronological age that the model can determine if that ECG parameter deviates from health at that age or not. In contrast, DNN-type Heart Age models have focused on predicting the actual chronological age of study participants.^15^

The A-ECG Heart Age was derived using strictly healthy individuals in whom cardiovascular risk factors or disease were actively ruled out. Hence, no disease signatures were embedded in the ECGs used for training the model beyond the ECG changes due to healthy aging. Beyond scientific curiosity, there appears to be little clinical relevance in predicting the actual chronological age from an ECG among patients with known or suspected cardiovascular disease. Interestingly, the association between Heart Age gap and the primary outcome was stronger in younger individuals who should otherwise be at lower risk of adverse cardiovascular events. In younger individuals, the association between Heart Age gap and prognosis is consistent with this association being attributable to risk factors and disease. In contrast, among older individuals, it is possible that a higher prevalence of other risk factors or established disease lessen the prognostic impact of the Heart Age gap.

The findings of the current study align with multiple previous publications on electrocardiographic signals of accelerated aging. The emerging understanding from DNA methylation studies of the process of accelerated cellular senescence in response to adverse lifestyle and external factors indicate that perhaps the ECG can be an accessible and intuitive ‘epigenetic clock’ with clinical and public health utility.^49–52^ This theory is further supported by findings of a weak albeit linear relationship between accelerated aging as measured using DNA methylation-based aging biomarkers and A-ECG Heart Age.^53^

A higher ECG Heart Age gap has been shown to be associated with Lamin-related gene (LMNA gene) mutations related to accelerated aging in Progeria syndrome,^38^ as well as impaired peripheral microvascular endothelial dysfunction, which is associated with vascular aging.^37^ Based on these observations, the association between the Heart Age gap and cardiovascular morbidity and mortality possibly stems from detecting subtle electrophysiological signatures related to accelerated cellular senescence.

The greater purpose of Heart Age gap is to have easy access to a simple yet prognostic and explainable biomarker of cardiovascular risk that can be easily deployed anywhere standard ECGs are performed, and intuitively communicated to a patient to aid personalized decision-making and intervention. For example, informing an individual with unaddressed risk factors that their heart is 15 years older than their chronological age could be a powerful message to spur preventive action. Similar attempts have been made in other areas, such as to incentivize smoking cessation through age estimations based on pulmonary function testing results.^54^

Beyond the mechanistic insights offered by the Heart Age gap, it extends its use to a more diverse population, particularly those in non-sinus rhythm. Whether Heart Age estimations in patients in atrial fibrillation can improve thromboembolic risk stratification merits testing in future studies.

The current Heart Age gap is the first Heart Age gap specifically developed for applications in non-sinus rhythms. Non-sinus rhythms including atrial fibrillation are a widely heterogeneous condition, and the potential applications of the Heart Age gap in these settings include phenotyping,^55^ risk stratification at time of diagnosis, and monitoring the effects of therapy. Such future studies of these applications are both justified and currently underway.

## Limitations

The validation cohort consisted of patients referred for CMR imaging, representing a group with a higher prevalence of cardiovascular risk factors and diseases compared with an otherwise relatively asymptomatic general practice outpatient population who could be a target for clinical application of the Heart Age gap. Despite this, the Heart Age gap retained its simplicity and utility even in this higher-risk group. Additionally, the cardiovascular risk factors and conditions observed in this cohort are representative of the very issues that preventive healthcare measures strive to address.

The combined clinical outcome for the validation group included death or hospitalization for heart failure, but not, for example, myocardial infarction, revascularization, or stroke. This is due to the nature of the available outcomes data. Future studies are justified to determine the prognostic association between A-ECG Heart Age gap and further cardiovascular outcomes.

The aim of the study was to derive an A-ECG Heart Age that can be applied to non-sinus rhythm and validate it internally and externally against previously published cohorts. Testing the non-P A-ECG Heart Age in cohorts with non-sinus rhythms is beyond the scope of the current study and is currently the subject of continued research.

## Conclusions

Accelerated cardiovascular aging with prognostic implications can be estimated accurately through A-ECG analysis applied to standard 10-s resting 12-lead ECGs even while excluding all P-wave-derived measures. We also mechanistically show that age and accelerated aging-related information is apparently robustly encoded in the ECG in domains beyond the P-wave. This study extends the applicability of A-ECG Heart Age estimations to patients who are in non-sinus rhythms and paves the way for further ECG-based risk stratification and phenotyping research in these rhythms, and such research is underway.

## Supplementary Material

ztae075_Supplementary_Data

## Data Availability

De-identified data of this study can be made available upon reasonable request to the corresponding author. Proposals for use will be reviewed on the basis of the scientific objective. A data use agreement will be required before the release of any raw results.
