# Supplementary material for: Advanced electrocardiography heart age: a prognostic, explainable machine learning approach applicable to sinus and non-sinus rhythms
Source: Eur Heart J Digit Health. 2024 Oct 9;6(1):45–54. doi: 10.1093/ehjdh/ztae075 (PMC11750191; doi:10.1093/ehjdh/ztae075)
Supplement: ztae075_Supplementary_Data [file ztae075_supplementary_data.docx]

**Advanced ECG Parameters**

| PARAMETER | EXPLANATION |
| --- | --- |
| 3D Tmax Azi | The azimuth in degrees of the spatial maximum T vector (see Rubulis A, et a, Heart Rhythm, 2004) |
| 3D Tmax Elv | The elevation in degrees of the spatial maximum T vector (see Rubulis A, et a, Heart Rhythm, 2004) |
| Limb sum | The sum in microvolts of the amplitudes of the QRS complexes (peak to trough) in the six limb leads. |
| RMS Tavplan | An expression of the "bulginess" of the T-wave loop defined as the mean distance in microvolts from the preferential plane for the sample values of the T loop. This is performed using an RMS calculation for this, whereas the original publishers of this technique (Nowinski K, et al, J Intern Med, 2000; Rubulis A, et al, Heart Rhythm, 2004) use a slightly different method. |
| SAI QRST | The summed absolute integral of the QRST, also called the sum absolute QRST integral, in units of mV*seconds, defined as the arithmetic sum of areas under the QRST curve in the Frank XYZ-leads' QRST signal averages (see Tereshchenko LG, et al, J Electrocardiol. 2010 Nov-Dec;43(6):548-52). This parameter differs from the spatial ventricular gradient in that the absolute areas under the QRST curves below baseline are added to, and not subtracted from, the areas above baseline. |
| T sv1 | The amplitude in microvolts of the first singular value of the T-wave derived from SVD of the 3-channel (derived Frank XYZ lead) signal-averaged T waves. |
| T sv2 | The amplitude in microvolts of the second singular value of the T-wave derived from SVD of the 3-channel (derived Frank XYZ lead) signal-averaged T waves. |
| T sv3 | The amplitude in microvolts of the third singular value of the T-wave derived from SVD of the 3-channel (derived Frank XYZ lead) signal-averaged T waves. |
| Xabs P area | The absolute amount of area subtended by the P wave in derived lead X, in units of microvolts*milliseconds |
| Xabs QRS area | The absolute amount of area subtended by the QRS wave in derived lead X, in units of microvolts*milliseconds |
| Xabs T area | The absolute amount of area subtended by the T wave in derived lead X, in units of microvolts*milliseconds |
| Xnet P area | The net area subtended by the P wave in derived lead X, in units of microvolts*milliseconds. This is the net area left over after subtracting out the amount of area below the isoelectric line from the total or absolute area. |
| Xnet QRS area | The net area subtended by the QRS wave in derived lead X, in units of microvolts*milliseconds. This is the net area left over after subtracting out the amount of area below the isoelectric line from the total or absolute area. |
| Xnet T area | The net area subtended by the T wave in derived lead X, in units of microvolts*milliseconds. This is the net area left over after subtracting out the amount of area below the isoelectric line from the total or absolute area. |
| Yabs P area | The absolute amount of area subtended by the P wave in derived lead Y, in units of microvolts*milliseconds |
| Yabs QRS area | The absolute amount of area subtended by the QRS wave in derived lead Y, in units of microvolts*milliseconds |
| Yabs T area | The absolute amount of area subtended by the T wave in derived lead Y, in units of microvolts*milliseconds |
| Ynet P area | The net area subtended by the P wave in derived lead Y, in units of microvolts*milliseconds. This is the net area left over after subtracting out the amount of area below the isoelectric line from the total or absolute area. |
| Ynet QRS area | The net area subtended by the QRS wave in derived lead Y, in units of microvolts*milliseconds. This is the net area left over after subtracting out the amount of area below the isoelectric line from the total or absolute area. |
| Ynet T area | The net area subtended by the T wave in derived lead Y, in units of microvolts*milliseconds. This is the net area left over after subtracting out the amount of area below the isoelectric line from the total or absolute area. |
| Zabs P area | The absolute amount of area subtended by the P wave in derived lead Z, in units of microvolts*milliseconds |
| Zabs QRS area | The absolute amount of area subtended by the QRS wave in derived lead Z, in units of microvolts*milliseconds |
| Zabs T area | The absolute amount of area subtended by the T wave in derived lead Z, in units of microvolts*milliseconds |
| Znet P area | The net area subtended by the P wave in derived lead Z, in units of microvolts*milliseconds. This is the net area left over after subtracting out the amount of area below the isoelectric line from the total or absolute area. |
| Znet QRS area | The net area subtended by the QRS wave in derived lead Z, in units of microvolts*milliseconds. This is the net area left over after subtracting out the amount of area below the isoelectric line from the total or absolute area. |
| Znet T area | The net area subtended by the T wave in derived lead Z, in units of microvolts*milliseconds. This is the net area left over after subtracting out the amount of area below the isoelectric line from the total or absolute area. |
| 12 Lead Area | The sum of the areas under the curves of the QRS complexes in the 12 standard leads, in units of mV*s. |
| 12 Lead Voltage | The absolute value of the QRS voltage in microvolts (R peaks to S troughs, or Q troughs to R peaks, whichever applies in each given lead) summed over all 12 conventional ECG leads. |
| 3D QRS 10 | The magnitude of the spatial QRS wave at 10 ms after its beginning, in units of microvolts |
| 3D QRS 20 | The magnitude of the spatial QRS wave at 20 ms after its beginning, in units of microvolts |
| 3D QRS 30 | The magnitude of the spatial QRS wave at 30 ms after its beginning, in units of microvolts |
| 3D QRS max | The maximum amplitude in microvolts of the 3-dimensional QRS loop, also referred to as the spatial peak QRS, spatial maximum QRS vector, or maximum QRS wave vector magnitude. |
| 3D T max mag | The maximum amplitude in microvolts of the 3-dimensional T loop, also referred to as the spatial peak T, or spatial maximum T-wave vector, or maximum T-wave vector magnitude. This is used along with the mean T-wave vector magnitude (3D T mean) to derive the so-called RMMV of the T, which is described in the section describing DEA below. |
| 3D T mean (and related RMMVT) | The mean T-wave vector magnitude in units of microvolts. This is used along with the maximum T-wave vector magnitude (see 3D Tmax mag above) to derive the RMMV of the T wave (RMMVT). |
| Beat# | Number to left side of decimal point is the # of accepted beats included in the QRS-T signal average, whereas number to right side of decimal point is the # of accepted beats included in the P signal average. |
| Cornell Product | The Cornell Voltage above multiplied by the QRS Duration above, units are mV*ms. |
| Cornell Voltage | The sum in mV of the absolute values of the voltage of R in lead aVL and the voltage of S in lead V3. |
| DEA (and related RMMVT) | The mean absolute value of the difference in degrees between the frontal plane T-wave elevation and azimuth of all T-wave loop samples (see Bortolan G and Christov I, CinC 28:633-636, 2001, and Batchvarov VN, et al, CinC 34:451-454, 2007). Note that the "RMMV" of T-wave of the same authors is simply herein the "3D Tmax mag" divided by the "3D T mean”. |
| e1 QRS-T | The angle in degrees between the first eigenvector (e1) of the QRS and the first eigenvector of the T. |
| e3 QRS-T | The angle in degrees between two other principal planes. Specifically, the first and second eigenvectors of the QRS form one plane, and the first and second eigenvectors of the T form a different plane. The third eigenvector (e3) is perpendicular to the plane formed by the first and second eigenvectors. Thus, this measurement (e3QRS-T) represents the angle between those two principal planes. |
| Eigen QRS a | The width in microvolts of the "broadside view" of the spatial QRS loop when that loop is projected into its own preferential space by using singular value decomposition. |
| Eigen QRS area | The area in millivolts squared of the "broadside view" spatial QRS loop when that loop is projected into its own preferential space by using singular value decomposition. |
| Eigen QRS b | The length in microvolts of the "broadside view" of the spatial QRS loop when that loop is projected into its own preferential space by using singular value decomposition. |
| Eigen QRS c | The width in microvolts of the "edgewise view" of the spatial QRS loop when that loop is projected into its own preferential space by using singular value decomposition. |
| Fr Lower L %% | The percent of the total QRS loop area in the frontal plane that subtends the left lower (left inferior) quadrant of that frontal plane. |
| Fr Lower R %% | The percent of the total QRS loop area in the frontal plane that subtends the right lower (right inferior) quadrant of that frontal plane. |
| Fr QRS 10 | The direction in degrees to which the QRS loop in the frontal plane points at 10 ms into that planar loop. |
| Fr QRS 20 | The direction in degrees to which the QRS loop in the frontal plane points at 20 ms into that planar loop. |
| Fr QRS 30 | The direction in degrees to which the QRS loop in the frontal plane points at 30 ms into that planar loop. |
| Fr QRS Max | The direction in degrees to which the QRS loop in the frontal plane points when that planar loop's voltage is at a maximum. |
| Fr T Max | The direction in degrees to which the T loop in the frontal plane points when that planar loop's voltage is at a maximum |
| Fr Upper L %% | The percent of the total QRS loop area in the frontal plane that subtends the left upper (left superior) quadrant of that frontal plane. |
| Fr Upper R %% | The percent of the total QRS loop area in the frontal plane that subtends the right upper (right superior) quadrant of that frontal plane. |
| Frontal Area | The total QRS loop area in the frontal plane in millivolts squared. |
| FrQRSmax | The maximum amplitude in microvolts of the frontal planar QRS loop. |
| Fujita R | The magnitude of only the purely rightward aspect of the maximal deflection of the QRS loop in the horizontal (transverse) plane, in units of microvolts (see Fujita K, et al, Jpn Circ J, 1976). |
| Hor Area | The total QRS loop area in the horizontal (transverse) plane in millivolts squared |
| Hor Lower L %% | The percent of the total QRS loop area in the horizontal (transverse) plane that subtends the left lower (patient's RIGHT anterior) quadrant of that horizontal plane |
| Hor Lower R %% | The percent of the total QRS loop area in the horizontal (transverse) plane that subtends the right lower (patient's LEFT anterior) quadrant of that horizontal plane |
| Hor QRS 10 | The direction in degrees to which the QRS loop in the horizontal (transverse) plane points at 10 ms into that planar loop |
| Hor QRS 20 | The direction in degrees to which the QRS loop in the horizontal (transverse) plane points at 20 ms into that planar loop |
| Hor QRS 30 | The direction in degrees to which the QRS loop in the horizontal (transverse) plane points at 30 ms into that planar loop |
| Hor QRS Max | The direction in degrees to which the QRS loop in the horizontal (transverse) plane points when that planar loop's voltage is at a maximum |
| Hor T Max | The direction in degrees to which the T loop in the horizontal (transverse) plane points when that planar loop's voltage is at a maximum |
| Hor Upper L %% | The percent of the total QRS loop area in the horizontal (transverse) plane that subtends the left upper (patient's RIGHT posterior) quadrant of that horizontal plane |
| Hor Upper R %% | The percent of the total QRS loop area in the horizontal (transverse) plane that subtends the right upper (patient's LEFT posterior) quadrant of that horizontal plane |
| HorQRSmax | The maximum amplitude in microvolts of the horizontal planar QRS loop |
| hr:min:sec | Length of the file in hr:min:sec |
| Jonas P wave morph type | See Holmqvist F, et al, BMC Cardiovasc Dis 2007 (named after senior author Jonas Carlson). Note: the method herein reflects a Kors' rather than inverse Dower VCG transform. |
| Maximum Angle (=spatial peaks QRS-T angle) | The angle in degrees in three-dimensional space between the maximum magnitude of the QRS loop and the T loop in the VCG derived from the standard 12-lead ECG using Kors’ transform. |
| Mean Angle (= spatial mean QRS-T angle) | The angle in degrees in three-dimensional space between the mean magnitude of direction for the QRS loop and the T loop in the VCG derived from the standard 12-lead ECG using Kors’ transform. |
| P Duration | The P duration interval in ms as derived from the spatial mean P wave rather than from the conventional ECG. |
| P Max SV | The maximum spatial velocity in mV/s of the derivative of the vector magnitude of the spatial P wave. Often invalid in the context of 50 Hz or 60 Hz noise. |
| P Mean SV | The average spatial velocity in mV/s of the derivative of the vector magnitude of the spatial P wave. Often invalid in the context of 50 Hz or 60 Hz noise. |
| P morph type | P-wave morph type (inspired by Jonas P wave morph type above), that also implements a threshhold criterion for better reproducibility/reliability. |
| P Nadir | The duration in ms from the start of the Frank XYZ-lead P-wave vector magnitude to the negative extreme (nadir value) within this same vector magnitude before the end of the P-wave. This phenomenon occurs because the baselines before and after the P-wave often are not the same. |
| P VM Integral | The integral or area under the curve of the unfiltered Frank XYZ -lead P vector magnitude in units of mV*s |
| P/A | The ratio of the maximum posteriorly directed QRS loop voltage to the maximum anteriorly directed QRS loop voltage in the horizontal plane |
| P/L | The ratio of the maximum posteriorly directed QRS loop voltage to the maximum leftwardly directed QRS loop voltage in the horizontal plane. See Fujita K, et al, Jpn Circ J, 1976. |
| PA/LR | The ratio of the "maximum posteriorly directed plus maximum anteriorly directed" QRS loop voltage to the "maximum leftwardly directed plus maximum rightwardly directed" QRS loop voltage in the horizontal plane. |
| Polar Vec Azi | The elevation angle in degrees of the polar vector. |
| Polar Vec Ele | The azimuth angle in degrees of the polar vector. |
| Polar Vec Mag | The magnitude of the polar vector. |
| PQ Interval | The PQ interval duration in ms as derived from spatial P-wave and spatial QRS-wave fiducial points rather than from the conventional P and QRS wave fiducial points |
| PR Angle | The spatial mean P-QRS angle in units of degrees |
| PSpatial Mean | The time-voltage in mV*s of the Frank XYZ-lead P vector magnitude (spatial mean P wave). |
| QRS area x | The time-voltage (area) in mV*s of the QRS waveform in lead X |
| QRS area y | The time-voltage (area) in mV*s of the QRS waveform in lead Y |
| QRS area z | The time-voltage (area) in mV*s of the QRS waveform in lead Z |
| QRS Azi 1/8 | The azimuth angle in degrees of the 3-dimensional QRS loop when one eighth of the way into the loop |
| QRS Azi 2/8 | The azimuth angle in degrees of the 3-dimensional QRS loop when two eighths of the way into the loop |
| QRS Azi 3/8 | The azimuth angle in degrees of the 3-dimensional QRS loop when three eighths of the way into the loop |
| QRS Azi 4/8 | The azimuth angle in degrees of the 3-dimensional QRS loop when four eighths of the way into the loop |
| QRS Azi 5/8 | The azimuth angle in degrees of the 3-dimensional QRS loop when five eighths of the way into the loop |
| QRS Azi 6/8 | The azimuth angle in degrees of the 3-dimensional QRS loop when six eighths of the way into the loop |
| QRS Azi 7/8 | The azimuth angle in degrees of the 3-dimensional QRS loop when seven eighths of the way into the loop |
| QRS Azi 8/8 | The azimuth angle in degrees of the 3-dimensional QRS loop when eight eighths of the way into the loop |
| QRS Duration | The QRS interval in ms as derived from the Frank-lead QRS vector magnitude (spatial QRS) rather than from the conventional ECG |
| QRS Elv 1/8 | The elevation angle in degrees of the 3-dimensional QRS loop when one eighth of the way into the loop |
| QRS Elv 2/8 | The elevation angle in degrees of the 3-dimensional QRS loop when two eighths of the way into the loop |
| QRS Elv 3/8 | The elevation angle in degrees of the 3-dimensional QRS loop when three eighths of the way into the loop |
| QRS Elv 4/8 | The elevation angle in degrees of the 3-dimensional QRS loop when four eighths of the way into the loop |
| QRS Elv 5/8 | The elevation angle in degrees of the 3-dimensional QRS loop when five eighths of the way into the loop |
| QRS Elv 6/8 | The elevation angle in degrees of the 3-dimensional QRS loop when six eighths of the way into the loop |
| QRS Elv 7/8 | The elevation angle in degrees of the 3-dimensional QRS loop when seven eighths of the way into the loop |
| QRS Elv 8/8 | The elevation angle in degrees of the 3-dimensional QRS loop when eight eighths of the way into the loop |
| QRS Mag 1/8 | The magnitude in microvolts of the 3-dimensional QRS loop when one eighth of the way into the loop |
| QRS Mag 2/8 | The magnitude in microvolts of the 3-dimensional QRS loop when two eighths of the way into the loop |
| QRS Mag 3/8 | The magnitude in microvolts of the 3-dimensional QRS loop when three eighths of the way into the loop |
| QRS Mag 4/8 | The magnitude in microvolts of the 3-dimensional QRS loop when four eighths of the way into the loop |
| QRS Mag 5/8 | The magnitude in microvolts of the 3-dimensional QRS loop when five eighths of the way into the loop |
| QRS Mag 6/8 | The magnitude in microvolts of the 3-dimensional QRS loop when six eighths of the way into the loop |
| QRS Mag 7/8 | The magnitude in microvolts of the 3-dimensional QRS loop when seven eighths of the way into the loop |
| QRS Mag 8/8 | The magnitude in microvolts of the 3-dimensional QRS loop when eight eighths of the way into the loop |
| QRS Max Right | The magnitude of the maximum overall "toward the right" deflection of the QRS loop in the horizontal (transverse) plane, in units of microvolts. This is akin to the hypotenuse, whereas the "Fujita R" and the "Fujita P" or "Fujita A" are akin to the sides, of the triangle describing the maximal rightward QRS deflection in the horizontal plane, that deflection either being right-posterior or right-anterior. |
| QRS Max SV | The maximum spatial velocity in mV/s of the derivative of the vector magnitude of the spatial QRS wave. |
| QRS Mean SV | The average spatial velocity in mV/s of the derivative of the vector magnitude of the spatial QRS wave. |
| QRS morph | An implementation of the “Jonas P wave morph type”, but applied to the QRS wave instead, and that also implements a threshhold criterion for better reproducibility/reliability. |
| QRS Ratio | One can attempt to find a two dimensional plane that maximizes the projection of the QRS loop onto two dimensions. The maximal projection of this loop can be divided into two areas by dividing the loop with a line segment from the origin of the loop to the peak of the loop (R). The QRS ratio is defined as the ratio of the area of the second half of the loop to the area of the first half. |
| QT Interval | The uncorrected QT interval in ms as derived from the spatial QRS and spatial T waves rather than from the conventional ECG. |
| Qx | The amplitude in mV of the Q wave in lead X |
| Qy | The amplitude in mV of the Q wave in lead Y |
| Qz | The amplitude in mV of the Q wave in lead Z |
| R/L | The ratio of the maximum rightwardly directed QRS loop voltage to the maximum leftwardly directed QRS loop voltage in the horizontal plane |
| RV6/SV1 | The unitless ratio of the R-wave voltage of conventional ECG lead V6 to the S-wave voltage of conventional ECG lead V1 |
| RV6/Rmax | The unitless ratio of the R-wave voltage of conventional ECG lead V6 to the R-wave voltage of that conventional ECG lead that has the largest voltage of all such conventional leads |
| RV6/SV6 | The unitless ratio of the R-wave voltage of conventional ECG lead V6 to the S-wave voltage of conventional ECG lead V6 |
| Rx | The amplitude in mV of the R wave in lead X |
| Ry | The amplitude in mV of the R wave in lead Y |
| Rz | The amplitude in mV of the R wave in lead Z |
| Rz Duration | The duration in ms of the R wave in the Frank Z-lead |
| Sag Lower L %% | The percent of the total QRS loop area in the left sagittal plane that subtends the left lower (anterior inferior) quadrant of that left sagittal plane |
| Sag Lower R %% | The percent of the total QRS loop area in the left sagittal plane that subtends the right lower (posterior inferior) quadrant of that left sagittal plane |
| Sag QRS 10 | The direction in degrees to which the QRS loop in the left sagittal plane points at 10 ms into that planar loop |
| Sag QRS 20 | The direction in degrees to which the QRS loop in the left sagittal plane points at 20 ms into that planar loop |
| Sag QRS 30 | The direction in degrees to which the QRS loop in the left sagittal plane points at 30 ms into that planar loop |
| Sag QRS Max | The direction in degrees to which the QRS loop in the left sagittal plane points when that planar loop's voltage is at a maximum |
| Sag T Max | The direction in degrees to which the T loop in the left sagittal plane points when that planar loop's voltage is at a maximum |
| Sag Upper L %% | The percent of the total QRS loop area in the left sagittal plane that subtends the left upper (anterior superior) quadrant of that left sagittal plane |
| Sag Upper R %% | The percent of the total QRS loop area in the left sagittal plane that subtends the right upper (posterior superior) quadrant of that left sagittal plane |
| Sagittal Area | The total QRS loop area in the left sagittal plane in millivolts squared |
| SagQRSmax | The maximum amplitude in microvolts of the left sagittal planar QRS loop |
| Sokolow-Lyon | The sum in mV of the absolute values of the voltage of S in lead V_1_ and the voltage of R in lead V_5_ or V_6_ whichever is greater. |
| Sp Mean T | The time-voltage in mV*s of the spatial mean T wave. |
| Spatial Mean QRS | The time-voltage in mV*s of the Frank-lead QRS vector magnitude (spatial mean QRS wave). |
| Spatial Vat | The spatial ventricular activation time (spatial intrinsicoid deflection) in ms. This is the time in ms from the beginning of the spatial QRS wave to the peak of the R wave of the same spatial QRS wave. See Ishizawa K, et al, Am Heart J, 1976, |
| ST Azi 1/8 | The azimuth angle in degrees of the 3-dimensional QRS loop when one eighth of the way into the loop, The ST segment loop extends from the end of the QRS wave to the end of the T wave. |
| ST Azi 2/8 | The azimuth angle in degrees of the 3-dimensional QRS loop when two eighths of the way into the loop. The ST segment loop extends from the end of the QRS wave to the end of the T wave. |
| ST Azi 3/8 | The azimuth angle in degrees of the 3-dimensional QRS loop when three eighths of the way into the loop. The ST segment loop extends from the end of the QRS wave to the end of the T wave. |
| ST Azi 4/8 | The azimuth angle in degrees of the 3-dimensional QRS loop when four eighths of the way into the loop. The ST segment loop extends from the end of the QRS wave to the end of the T wave. |
| ST Azi 5/8 | The azimuth angle in degrees of the 3-dimensional QRS loop when five eighths of the way into the loop. The ST segment loop extends from the end of the QRS wave to the end of the T wave. |
| ST Azi 6/8 | The azimuth angle in degrees of the 3-dimensional QRS loop when six eighths of the way into the loop. The ST segment loop extends from the end of the QRS wave to the end of the T wave. |
| ST Azi 7/8 | The azimuth angle in degrees of the 3-dimensional QRS loop when seven eighths of the way into the loop. The ST segment loop extends from the end of the QRS wave to the end of the T wave. |
| ST Azi 8/8 | The azimuth angle in degrees of the 3-dimensional QRS loop when eight eighths of the way into the loop. The ST segment loop extends from the end of the QRS wave to the end of the T wave. |
| ST Elv 1/8 | The elevation angle in degrees of the 3-dimensional QRS loop when one eighth of the way into the loop. The ST segment loop extends from the end of the QRS wave to the end of the T wave. |
| ST Elv 2/8 | The elevation angle in degrees of the 3-dimensional QRS loop when two eighths of the way into the loop. The ST segment loop extends from the end of the QRS wave to the end of the T wave. |
| ST Elv 3/8 | The elevation angle in degrees of the 3-dimensional QRS loop when three eighths of the way into the loop. The ST segment loop extends from the end of the QRS wave to the end of the T wave. |
| ST Elv 4/8 | The elevation angle in degrees of the 3-dimensional QRS loop when four eighths of the way into the loop. The ST segment loop extends from the end of the QRS wave to the end of the T wave. |
| ST Elv 5/8 | The elevation angle in degrees of the 3-dimensional QRS loop when five eighths of the way into the loop. The ST segment loop extends from the end of the QRS wave to the end of the T wave. |
| ST Elv 6/8 | The elevation angle in degrees of the 3-dimensional QRS loop when six eighths of the way into the loop. The ST segment loop extends from the end of the QRS wave to the end of the T wave. |
| ST Elv 7/8 | The elevation angle in degrees of the 3-dimensional QRS loop when seven eighths of the way into the loop. The ST segment loop extends from the end of the QRS wave to the end of the T wave. |
| ST Elv 8/8 | The elevation angle in degrees of the 3-dimensional QRS loop when eight eighths of the way into the loop. The ST segment loop extends from the end of the QRS wave to the end of the T wave. |
| ST Mag 1/8 | The magnitude in microvolts of the 3-dimensional ST segment loop when one eighth of the way into the loop. The ST segment loop extends from the end of the QRS wave to the end of the T wave. |
| ST Mag 2/8 | The magnitude in microvolts of the 3-dimensional ST segment loop when two eighths of the way into the loop. The ST segment loop extends from the end of the QRS wave to the end of the T wave. |
| ST Mag 3/8 | The magnitude in microvolts of the 3-dimensional ST segment loop when three eighths of the way into the loop. The ST segment loop extends from the end of the QRS wave to the end of the T wave. |
| ST Mag 4/8 | The magnitude in microvolts of the 3-dimensional ST segment loop when four eighths of the way into the loop. The ST segment loop extends from the end of the QRS wave to the end of the T wave. |
| ST Mag 5/8 | The magnitude in microvolts of the 3-dimensional ST segment loop when five eighths of the way into the loop. The ST segment loop extends from the end of the QRS wave to the end of the T wave. |
| ST Mag 6/8 | The magnitude in microvolts of the 3-dimensional ST segment loop when six eighths of the way into the loop. The ST segment loop extends from the end of the QRS wave to the end of the T wave. |
| ST Mag 7/8 | The magnitude in microvolts of the 3-dimensional ST segment loop when seven eighths of the way into the loop. The ST segment loop extends from the end of the QRS wave to the end of the T wave. |
| ST Mag 8/8 | The magnitude in microvolts of the 3-dimensional ST segment loop when eight eighths of the way into the loop. The ST segment loop extends from the end of the QRS wave to the end of the T wave. |
| Sx | The amplitude in mV of the S wave in lead X |
| Sy | The amplitude in mV of the S wave in lead Y |
| Sz | The amplitude in mV of the S wave in lead Z |
| T area x | The time-voltage (area) in mV*s of the T waveform in lead X |
| T area y | The time-voltage (area) in mV*s of the T waveform in lead Y |
| T area z | The time-voltage (area) in mV*s of the T waveform in lead Z |
| T Max SV | The maximum spatial velocity in mV/s of the derivative of the vector magnitude of the spatial T wave. Often invalid in the context of 50 Hz or 60 Hz noise. |
| T Mean SV | The average spatial velocity in mV/s of the derivative of the vector magnitude of the spatial T wave. Often invalid in the context of 50 Hz or 60 Hz noise |
| T morph | Inspired by “Jonas P wave morph type”, but applied to the T wave instead, and that also implements a threshhold criterion for better reproducibility/reliability |
| Teigenv | The quotient between the two highest T-loop eigenvalues (approximate diameters) of the T-wave "matrix of inertia". See Nowinski K, et al, J Intern Med, 2000; Rubulis A, et al, Heart Rhythm, 2004. I.e., #1/#2, which is the inverse of the more typical "PCA ratio". |
| TpTe | The duration in ms of the time from the peak till the time of the end of the Frank XYZ-lead T-wave vector magnitude ("spatial TpTe"). This is not a very useful parameter. |
| V G Frontal | The elevation angle in degrees of the spatial ventricular gradient as projected into the frontal (X vs -Y) plane |
| V G Hor | The elevation angle in degrees of the spatial ventricular gradient as projected into the horizontal (X vs Z = transverse) plane |
| V G Sagittal | The elevation angle in degrees of the spatial ventricular gradient as projected into the left sagittal (Z vs -Y) plane |
| Vent. Gradient | The time-voltage in mV*s of the spatial ventricular gradient (SVG). |
| VG area x | The time-voltage (area) in mV*s of the QRS plus T waveforms (ventricular gradient) in lead X |
| VG area y | The time-voltage (area) in mV*s of the QRS plus T waveforms (ventricular gradient) in lead Y |
| VG area z | The time-voltage (area) in mV*s of the QRS plus T waveforms (ventricular gradient) in lead Z |
| VG Fr Azi | The azimuth angle in degrees of the spatial ventricular gradient as projected into the frontal (X vs -Y) plane |
| VG Hor Azi | The azimuth angle in degrees of the spatial ventricular gradient as projected into the horizontal (X vs Z = transverse) plane |
| VG Sag Azi | The azimuth angle in degrees of the spatial ventricular gradient as projected into the left sagittal (Z vs -Y) plane |
| VM Integral | The integral or area under the curve of the 5-250 Hz filtered Frank XYZ -lead QRS vector magnitude in units of mV*s (default filter is 5-250 Hz FIR) |
| VM Max RMS | The maximum (peak) root mean square voltage in mV of the 5-250 Hz filtered Frank XYZ -lead QRS vector magnitude (default filter is 5-250 Hz FIR) |
| VM Total RMS | The total root mean square voltage in mV of the 5-250 Hz filtered Frank XYZ-lead QRS vector magnitude (default filter is 5-250 Hz FIR) |
| Z Integral | The integral or area under the curve of the 5-250 Hz filtered Frank Z-lead QRS complex in units of mV*s (default filter is 5-250 Hz FIR) |
| Z Max RMS | The maximum (peak) root mean square voltage in mV of the 5-250 Hz filtered Frank Z -lead QRS complex (default filter is 5-250 Hz FIR) |
| Z Total RMS | The total root mean square voltage in mV of the 5-250 Hz filtered Frank Z -lead QRS complex (default filter is 5-250 Hz FIR). |
